# Methods of 10-Meter Walk Test and Repercussions for Reliability Obtained in Typically Developing Children

**DOI:** 10.1155/2020/4209812

**Published:** 2020-08-20

**Authors:** Cyntia R. J. A. de Baptista, Amanda M. Vicente, Mariana A. Souza, Juliana Cardoso, Vanessa M. Ramalho, Ana C. Mattiello-Sverzut

**Affiliations:** ^1^Department of Health Science, Ribeirão Preto Medical School, University of São Paulo, Ribeirão Preto 14049-900, Brazil; ^2^Rehabilitation and Functional Performance Graduate Program, Ribeirão Preto Medical School, University of São Paulo, Ribeirão Preto 14049-900, Brazil

## Abstract

**Introduction:**

Research and clinical settings use the 10-meter walk test (10MWT) to measure locomotor capacity with considerable methodological diversity. Comparison between healthy and disabled children is frequent; however, the reproducibility of 10MWT using different methods is unknown.

**Objectives:**

This study analysed intrasubject, test-retest reliability, and agreement of four methods of 10MWT, exploring the influence of pace, acceleration-deceleration phases, and anthropometric measurements when calculating mean velocity.

**Methods:**

This cross-sectional study evaluated 120 typical children, both sexes, aged 6, 8, 10, and 12 (*n* = 30 for each age). The mean times and velocities of the path (10 m) and middle path (6 m) obtained at a self-selected and fast pace were analysed. Initial assessment and another after seven days recorded three measurements per method (sV6 = self-selected pace and 6 m; sV10 = self-selected pace and 10 m; fV6 = fast pace and 6 m; fV10 = fast pace and 10 m). Interclass correlation coefficient (ICC), multiple regression, and Snedecor-F test (5% significance level) were used.

**Results:**

The fV10 method had high intrasubject reliability for all tested ages (0.70 < ICC > 0.89); sV10 exhibited high intrasubject reliability for ages 6, 8, and 12 (0.70 < ICC > 0.89) and moderate for age 10 (0.50 < ICC < 0.69).Test-retest reliability at sV6 and fV6 did not reach high ICC in any tested ages. The test-retest reliability at sV10 and fV10 was moderate for ages 6, 8, and 12 (0.50 < ICC > 0.69) and poor for age 10 (0.25 < ICC > 0.49). There was no agreement between methods: sV6 versus sV10 (mean difference = 0.91 m/s; SEM = 0.036); fV6 versus fV10 (mean difference = 1.70; SEM = 0.046). The fV6 method versus fV10 overestimated the velocity (bias = 1.70 m/s).

**Conclusions:**

For typical children, the method that ensured the highest intrasubject reliability used fast pace and 10 m. Moreover, test-retest reliability increased when adopting 10 m at both self-selected and fast pace. The methods were not equivalent but were related, and those that did not compute the entire pathway overestimated the results.

## 1. Introduction

The 10-meter walk test (10MWT) is a simple assessment to measure locomotor capacity in clinical and research settings. Outcome measures originally recommended are the time taken to complete the test [1] or the mean velocity [2, 3]. The mean velocity of gait has been termed the sixth vital sign [4] because of its clinical and research relevance.

The 10MWT has measured locomotor capacities in adults [5, 6] and children [7–11] with several neuromotor diseases. However, the operational procedures can influence the outcome of the 10MWT, as shown in adults [12, 13]. Similarly, there is methodological diversity in obtaining 10MWT in the paediatric population [14–18]. Different methods used to obtain outcome measures, such as standing start, walking start, self-selected pace, fast pace, and automatic or manual stopwatch system make the comparison of results between studies challenging, especially concerning the distance used for timing of the test and the pace allowed. Besides, there is a limited number of studies in children with typical development and growth [19, 20], and there is no information on the reliability of 10MWT.

The 10MWT reliability studies focused on the paediatric population with neuromotor alterations show good validity, clinical significance [7, 21], and good intraexaminer and interexaminer reliability [22] in protocols using varying paces, distances, and commands. When different paces were requested, children and adolescents with neurological dysfunctions exhibited high reliability in evaluations obtained at self-selected velocity when compared to those performed at a fast velocity [15, 21]. For typically developing children and adolescents, the reliability of 10MWT at different test velocities is not known. A study of 350 healthy participants aged 2 to 12 years has normative values for timed tests, including 10MWT [19], but the study does not explore the psychometric properties.

There is a lack of consensus about methods of obtaining the 10MWT, as well as the need for its reliability data for the paediatric population.

The primary aim of this study was to analyse the reliability and agreement of 10MWT in terms of mean gait velocity (intrarater and test-retest reliability) by typical children from 6 to 12 years old when adopting two commands (self-selected and fast velocity) and two paths (6 and 10 meters).

As a secondary objective, the study compared the mean velocity developed in the 10 m and 6 m distance and explored how this outcome is influenced by acceleration-deceleration phases and anthropometric measurements such as height, mass, lower limb length, and quadriceps angle.

## 2. Materials and Methods

This observational, cross-sectional, descriptive study designed to test the reliability and agreement of 10MWT evaluated 120 participants aged 6, 8, 10, and 12 years old and of both sexes.

Inclusion criteria were belonging to the target ages of the study. Exclusion criteria were having a history of fracture of the lower limbs and pelvis, deformities, and diseases affecting walking, not understanding the commands of an evaluator, and using a walking aid, prosthesis, and orthosis, or insoles.

Personal data, weight, height, real length (distance from the anterior superior iliac spine to ipsilateral medial malleolus), and apparent length (distance from umbilical scar to ipsilateral medial malleolus) of the lower limbs and quadriceps angle were recorded for participants who met inclusion criteria. Anthropometric measurements were obtained in the standing position, so as not to ignore the effect of weight-bearing on lower limb alignment. A tape was used to measure lower limbs' length [23]. A goniometer was used to measure the quadriceps angle (the angle formed by a line from the anterior superior iliac spine to the patella centre and a line from the patella centre to the tibial tuberosity with the participant in standing position with relaxed limbs) [24].

**Figure 1:**
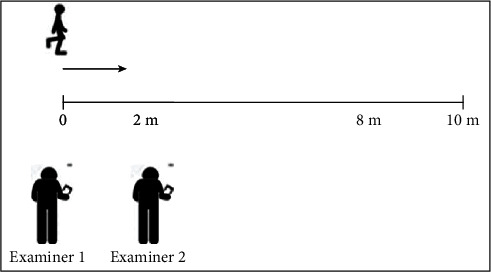
10MWT path representation.

Participants underwent testing for the 10MWT at two times: admission (test) and after seven days (retest). The participants did the10MWT on a regular, flat surface sports gymnasium with the start and endpoints of the path marked on the floor for viewing by the examiners. Participants performed the test barefoot, adopting normal base of support and arms by the sides as the starting position. Participants had undergone a previous familiarization test. The standardized verbal command was “1, 2, 3, go!” Each test condition had three times records, using self-selected velocity associated with 6 m (sV6) and 10 m (sV10) computation; and fast velocity associated with the computation of 6 m (fV6) and 10 m (fV10) of the path. In the self-selected velocity test (sV6 and sV10), the periodic verbal instruction to participants was “Walk without running, as you walk every day.” At fast velocity (fV6 and fV10), the verbal instruction periodically was “go fast but not running.” To evaluate the influence of acceleration-deceleration phases, the 10MWT was timed (Chronobio Stopwatch SW2018) simultaneously by two examiners. Examiner 1 recorded the time taken to cover the total distance (10 m), and the collected data represent the standing start mode. Examiner 2 recorded the time to cover 6 m (disregarding the initial 2 m and the final 2 m of the path) without participant was aware of the limits of this path (Figure 1).

Statistical analysis used SAS statistical software (version 9.3; SAS Institute Inc., Cary, NC) and SPSS software (version 17.0), adopting a 5% significance level. The outcome variable was the mean velocity of the 10MWT. Interclass correlation coefficient (ICC 2.1) assessed the reliability of measurements in self-selected velocity, fast velocity, middle path (6 m), total path (10 m), and test-retest reliability (ICC 3.k) [25]. The reliability was classified as poor (ICC<0.25); low (0.26 < ICC < 0.49); moderate (0.50 < ICC < 0.69); high (0.70-0.89); and extremely high (0.90-1.0). Bland-Altman plots were used to analyse the agreement between different paces and distances (sV6 and sV10; fV6 and fV10). The influence of anthropometric variables on 10MWT was analysed by multiple regression, considering self-selected and fast velocities as dependent variables and weight, height, real lower limb length (RL), apparent lower limb length (AL), and quadricipital angle (QA) as independent variables. Snedecor *F* test analysed the variability of sV6, sV10, fV6, and fV10.

## 3. Results

### 3.1. Sample Characterization

All analyses presented used the right lower limb data for boys and girls together, as there was no difference between sexes or right and left anthropometric measures (RL, AL, QA) (*p* > 0.05). Intrasubject reliability analysis included 120 participants (*n* = 30; aged 6, 8, 10, and 12 years) while test-retest reliability included 83 participants (*n* = 21 aged 6; *n* = 20 aged 8; *n* = 20 aged 10; *n* = 22 aged 12) because adherence to retest was partial.  Table 1 presents the mean and standard deviations of the anthropometric variables.

### 3.2. Intrasubject and Test-Retest Reliability

The three 10MWT trials per participant performed on sV6 demonstrated high intrasubject reliability for 6, 8, and 12 year olds (0.70 < ICC > 0.89) and moderate for 10 year olds (0.50 < ICC > 0.69) (Table 2). When performed on fV6, there was high intrasubject reliability for ages 8 and 10 year olds (0.70 < ICC > 0.89) and moderate for ages 6 and 12 (0.50 < ICC > 0.69) (Table 2).

When performed on sV10, velocity data demonstrated high intrasubject reliability for 6, 8, and 12 year olds (0.70 < ICC > 0.89) and moderate for 10 year olds (0.50<ICC>0.69) (Table 2). When performed on fV10, velocity data exhibited high reliability for all ages (0.70 < ICC > 0.89) (Table 2).

The mean velocity to cover 10MWT under sV6 condition exhibited moderate test-retest reliability for 8 and 12 year olds (0.50 < ICC > 0.69) and low for 6 and 10 year olds (0.26 < ICC < 0.49) (Table 2). Under the fV6 condition, test-retest reliability was moderate for 12 year olds participants (0.50 < ICC > 0.69) and low for 6, 8, and 10 year olds (0.26 < ICC < 0.49) (Table 2).

The mean velocity to cover 10MWT under sV10 condition exhibited moderate test-retest reliability for 6, 8, and 12 year olds (0.50 < ICC > 0.69) and low for 10 year olds (0.26 < ICC < 0.49) (Table 2). Under the fV10 condition, test-retest reliability was moderate for 6-, 8- and 12-year-old participants (0.50 < ICC > 0.69) and low for 10 year olds (0.26 < ICC < 0.49) (Table 2).

### 3.3. Agreement between Conditions (sV6 and sV10, fV6 and fV10)

**Figure 2:**
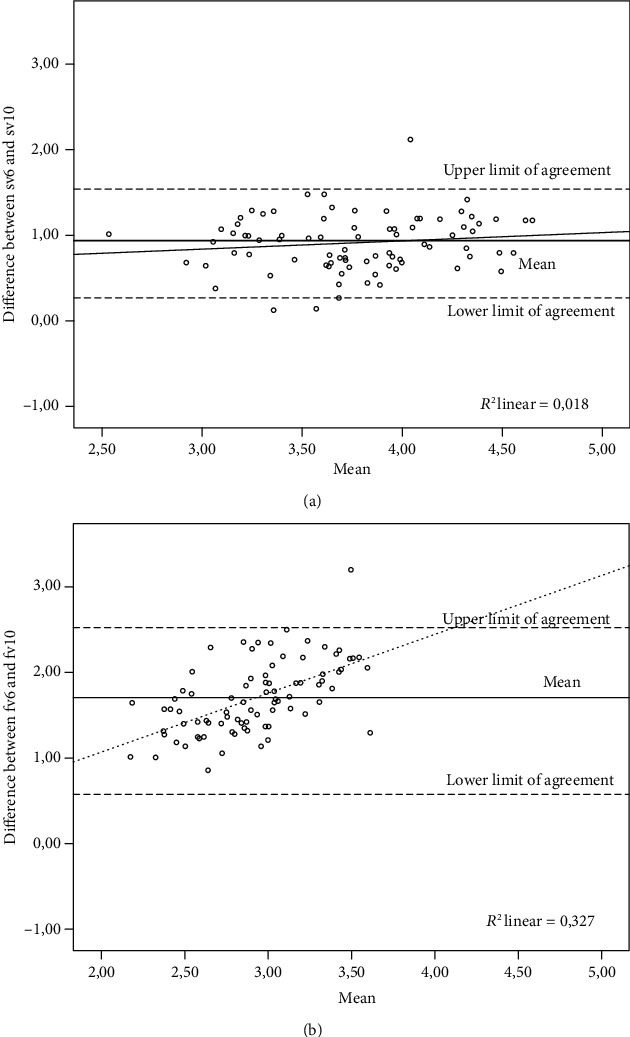
Bland-Altman plot of sV6 versus sV10 (a), fV6 versus fV10 (b). Continuous lines represent the mean difference (systematic bias); lines build by dots represent (proportional bias); noncontinuous lines represent the upper and lower limits of agreement.

There was a significant difference between the mean velocity of sV6 and sV10 (mean difference = 0.91; SD = 0.33; upper limit = 1.55; lower limit = 0.27; SEM = 0.036, *p* < 0.05) and fV6 versus fV10 (mean difference = 1.70; SD = 0.42; upper limit = 2.52; lower limit = 0.88; SEM = 0.046, *p* < 0.05). Bland-Altman plots showed an overestimation of the self-selected velocity condition of sV6 when compared to sV10 (systematic bias) (Figure 2(a)). Similarly, there was an overestimation of mean velocity at fV6 when compared to fV10 (bias = 1.70 m/s) and a trend (proportional bias) to increase with the increasing magnitude of velocity (Figure 2(b)).

### 3.4. Influence of Anthropometric Variables

Among the anthropometric variables studied, height significantly influenced the mean velocity of 10MWT in the sV10 condition (beta = 0.997) (Table 3).

Table 4 presents the comparisons between sV6 and sV10, fV6, and fV10, to analyse the influence of the distance used in the calculation of the mean velocity of 10MWT, based on the ratio between variances (Snedecor *F* test). It is noteworthy that this analysis used the baseline data (*n* = 30 by age). Self-selected pace tests did not show significant differences in the variances of the mean velocity developed at 6 m and 10 m (*p* < 0.05) (Table 4). In contrast, tests performed at a fast pace showed significant differences between the mean velocities calculated at 6 m and 10 m for children aged 6, 10, and 12 years. The highest ratio found was in 12-year-old children (Table 4).

## 4. Discussion

The present study allowed verification of the satisfactory reliability of 10MWT among children with typical development when it recorded different paces (self-selected and fast paces) and paths (6 m and 10 m). When the 10MWT was performed at a fast pace, mean velocities calculated at 6 m and 10 m were significantly different, confirming the influence of acceleration and deceleration. Additionally, this study confirmed that among the anthropometric variables studied, height significantly influences the results of 10MWT in children, especially when the test is done as a self-selected pace over a 10 m distance to calculate mean velocity.

Overall, there was high or moderate intrasubject reliability of 10 MWT for all ages (ICC from 0.70 to 0.89), and the mean velocity in the fV10 condition is similar to those obtained in studies with typical children and adolescents [19, 26]. There is a lack of comparable results about reliability in the literature involving typical children. In the case of cerebral palsy, higher reliability occurred in self-selected velocity tests when compared to those performed at fast velocity [21] as the difficulties in motor control inherent to the disease lead to high variability in fast walking [27].

In the case of our study, typical children assessed at their natural pace have similar variances between the 6 m and 10 m distances (variance ratios, Table 4), except for those 10 year olds (Table 4). However, under the command of walking at a fast velocity, there was high gait variability for most children (6, 8, and 12 year olds) (Table 4). Also, under the command of walking at a fast velocity, our children developed higher mean velocity in the intermediate section (6 m), a result expressed by the variance ratios being significantly higher than 1 for most age groups (6, 10, and 12 year olds). These data suggest that the 10MWT of typical children is minimally influenced by the acceleration and deceleration phases of gait if it is obtained at a self-selected velocity. In contrast, when applied at a fast velocity, 10MWT is more strongly influenced by the acceleration and deceleration phases when comparing 10 m and 6 m distances.

As for test-retest reliability, data from the present study showed that the velocities tested are similarly reliable if the evaluator adopts total distance (10 m). In this last aspect (distance used), the test-retest reliability was similar to that obtained in children with neuromotor diseases [15]. When computing the entire test path (10 m) and without participants walking at a fast pace, the velocity data showed high reliability.

By associating the findings of our study with the prior knowledge that gait speed becomes more consistent as the child grows, matures and has gross motor coordination [28], it is possible to point out some recommendations regarding 10MWT. The best methods were those involving most of the age groups (6, 8, 12 years) and satisfactory reliability, the condition that used the10 m path was better than 6 m, regardless of the pace requested. The worst results were with the combination of fast velocity and the intermediate distance (6 m) with most of the groups presenting poor reliability (6, 8, and 12 year olds). Faced with the absence of similar results in the literature involving typical children, we compared our study with those performed in children with neuromotor diseases [15, 18, 21]. The theme is still controversial, as there are studies involving children with neuromotor diseases that report high [18] and low 10MWT test-retest reliability obtained at a fast velocity [21]. It seems that in children with neuromotor diseases, fast velocity 10MWT tends to require considerable gross motor coordination. On the other hand, self-selected walking velocity better reflects the real pace used in everyday life for these participants.

The limitations of this study are related to the understanding of the test commands provided by two different evaluators. Fortunately, ICC values were satisfactory in younger age groups (6 and 8 years). Although these are healthy children, it is possible that some interference of understanding is possible since no test was applied to assess this construct. Similarly, the motivation level of the participants was not evaluated, and this factor can impact reliability results, as highlighted by Graser and colleagues (2016) [21]. Specifically, some results found at the age of 10 years showed poor test-retest reliability with no apparent cause.

The data in this study are clinically relevant because they address locomotor capacity and indicate which 10MWT methods are sufficiently reliable in typical children. Thus, clinicians and researchers can compare the performance of typically developing children with others affected by various diseases, knowing the 10MWT commands and distance measurements that demonstrate greater reliability.

## 5. Conclusion

The reliability of 10MWT for typical children is condition-specific. Analysed under different test conditions, 10MWT demonstrated high to moderate intrasubject reliability. Regarding the test-retest, performance at the 10 m distance presented satisfactory reliability but not for the age group of 10-year-old age group. Therefore, comparing the velocity of 10MWT of children at a single moment, the condition with a highest reliability is performing at a fast velocity using the entire 10 m path. Moreover, when it comes to test-retest reliability, the 10MWT can be performed at both self-selected and fast velocities if using the entire 10 m distance to calculate mean velocity.

## Figures and Tables

**Table 1:** Anthropometric variables.

	Age			
	6 (*n* = 30)	8 (*n* = 30)	10 (*n* = 30)	12 (*n* = 30)
Weight	24.4 (5.87)	27.83 (5.54)	37.67 (10.10)	51.2 (12.80)
Height	1.21 (0.06)	1.31 (0.05)	1.41 (0.07)	1.57 (0.10)
RL (right side)	60.92 (3.44)	67.67 (3.47)	74.28 (5.03)	84.33 (6.99)
RL (left side)	60.71 (3.56)	67.72 (71.73)	74.21 (4.71)	83.95 (6.83)
AL (right side)	65.17 (4.05)	71.73 (3.58)	79.09 (4.87)	89.07 (7.19)
AL (left side)	65.23 (4.13)	71.85 (3.91)	79.01 (4.73)	89.22 (7.09)
QA (right side)	9.90 (3.2)	10.27 (3.43)	12.97 (4.68)	11.67 (4.47)
QA (left side)	9.30 (2.84)	9.83 (3.77)	11.47 (4.31)	10.87 (3.19)

Mean (Standard deviation, SD); RL: real length of lower limb; AL: apparent length of lower limb; QA: quadricipital angle.

**Table 2:** Intrasubject and test-retest reliability, time, the mean velocity of 10MWT under sV6, fV6, sV10, and fV10 conditions.

Intrasubject reliability (3 trials) (*n* = 120)	Age (years)	Mean time (s)	Mean velocity (m/s)	ICC	CI 95%	*p* value
sV6	6	4.97	1.25	0.78	(0.65; 0.88)	0.001
	8	4.66	1.32	0.88	(0.79; 0.94)	0.001
	10	4.57	1.34	0.68	(0.51; 0.82)	0.001
	12	4.16	1.48	0.79	(0.65; 0.88)	0.001
	All	4.56	2.26	0.93	(0.90; 0.95)	0.001
fV6	6	3.05	2.00	0.51	(0.28; 0.69)	0.001
	8	2.75	2.23	0.72	(0.56; 0.84)	0.001
	10	2.67	2.30	0.78	(0.63; 0.88)	0.001
	12	2.58	2.36	0.62	(0.42; 0.78)	0.001
	All	2.71	3.74	0.88	(0.84;0.91)	0.001
sV10	6	8.68	1.20	0.86	(0.75; 0.92)	0.001
	8	8.20	1.22	0.83	(0.72; 0.91)	0.001
	10	8.04	1.30	0.68	(0.50; 0.82)	0.001
	12	7.36	1.39	0.88	(0.80; 0.94)	0.001
	All	8.03	1.28	0.84	(0.79;0.88)	0.001
fV10	6	5.59	1.82	0.76	(0.62; 0.87)	0.001
	8	5.15	1.98	0.75	(0.60; 0.86)	0.001
	10	4.93	2.06	0.82	(0.70; 0.90)	0.001
	12	4.07	2.15	0.72	(0.56; 0.84)	0.001
	All	4.99	2.03	0.93	(0.90; 0.95)	0.001
Test-retest (*n* = 83)	Age (years)	Mean time (s)	Mean velocity (m/s)	ICC	CI 95%	*p* value
sV6	6	4.82	2,07	0.48	(0.26; 0.65)	0.001
	8	4.59	2.18	0.66	(0.49; 0.78)	0.001
	10	4.15	2.41	0.29	(0.03; 0.51)	0.001
	12	4.08	2,45	0.65	(0.48; 0.77)	0.001
	All	4.38	2.28	0.64	(0.44;0.77)	0.001
fV6	6	3.01	3.32	0.42	(0.19; 0.60)	0.001
	8	2.72	3.68	0.35	(0.11; 0.55)	0.001
	10	2.42	4.12	0.41	(0.18; 0.60)	0.001
	12	2.56	3.91	0.51	(0.31; 0.67)	0.001
	All	2.66	3.75	0.65	(0.46;0.77)	0.001
sV10	6	8,49	1,18	0.55	(0.35; 0.70)	0.001
	8	7,96	1,26	0.62	(0.43; 0.75)	0.001
	10	7,29	1,37	0.33	(0.04; 0.55)	0.001
	12	7,09	1,41	0.65	(0.48; 0.77)	0.001
	All	7.96	1.31	0.64	(0.44; 0.77)	0.001
fV10	6	5.56	1,80	0.63	(0.44; 0.76)	0.001
	8	4.94	2,03	0.50	(0.29; 0.67)	0.001
	10	4.43	2,26	0.41	(0.15; 0.61)	0.001
	12	4.57	2,19	0.57	(0.38; 0.71)	0.001
	All	4.82	2.07	0.77	(0.64; 0.85)	0.001

Legend: sV6: self-selected velocity and 6 m; fV6: fast velocity and 6 m; sV10: self-selected velocity and 10 m; fV10: fast velocity and 10 m; ICC: Interclass correlation coefficient; CI: confidence interval; *p* < 0.05.

**Table 3:** Estimated coefficients (beta) for each anthropometric variable tested.

Estimated coefficients (beta)				
Condition	sV_6_	sV_10_	fV_6_	fV_10_
Weight	-0.006	-0.004	-0.002	-0.002
Height	0.848	0.997^∗^	0.610	0.638
RL	0.002	0.000	0.008	0.002
AL	0.000	-0.004	0.000	0.004
QA	-0.008	-0.006	-0.008	-0.008

^∗^ = *p* < 0.05.

**Table 4:** Ratios of 10MWT velocity variances when performed under different conditions (sV6, sV10, fV6, and fV10) according to age groups.

Age	Variance ratio sV6/sV10	IC95% of the ratio	*p* value	Variance ratio fV6/fV10	IC95% of the ratio	*p* value
6	1.08	(0.71; 1.64)	0.71	1.72	(1.13; 2.62)	0.01
8	1.29	(0.85 1.96)	0.23	1.35	(0.89; 2.05)	0.16
10	1.49	(0.98; 2.26)	0.06	1.8	(1.18; 2.73)	0.01
12	1.37	(0.90; 2.09)	0.14	2.04	(1.34; 3.09)	0.01
All	1.30	(1.37;2.08)	0,01	1.62	(2.09; 3.16)	0.01

*p* < 0.05; CI: confidence Interval (Snedecor *F* test).

## Data Availability

The 10MWT data used to support the findings of this study are included within the supplementary information file (supplementary file_10MWT).

## Supplementary Materials

Supplementary materials 10MWT datasets.
